# Supplementary material for: Laparoscopic Surgery with Concomitant Hernia Repair and Cholecystectomy: An Alternative Approach to Everyday Practice
Source: Diseases. 2023 Mar 3;11(1):44. doi: 10.3390/diseases11010044 (PMC10047121; doi:10.3390/diseases11010044)
Supplement: Supplementary file 1 [file diseases-11-00044-s001.zip › diseases-2153312-supplementary.pdf]

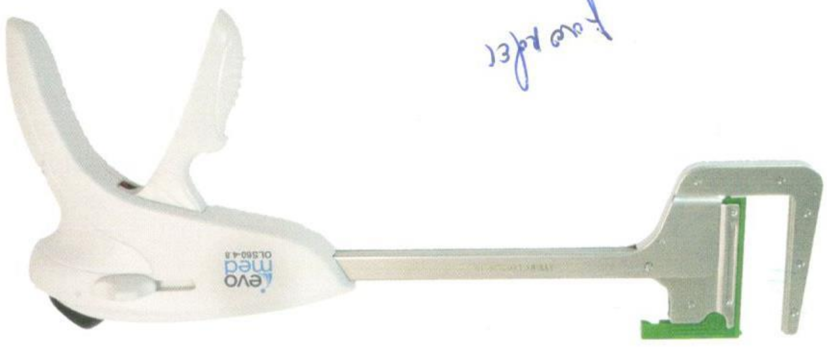

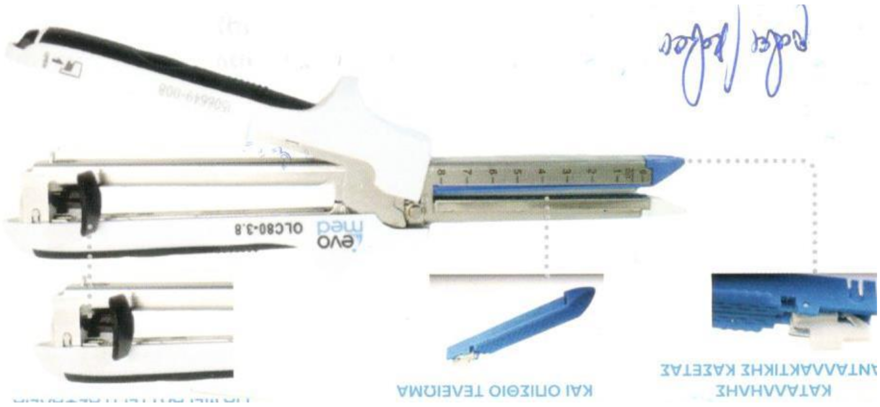

Laparascopica

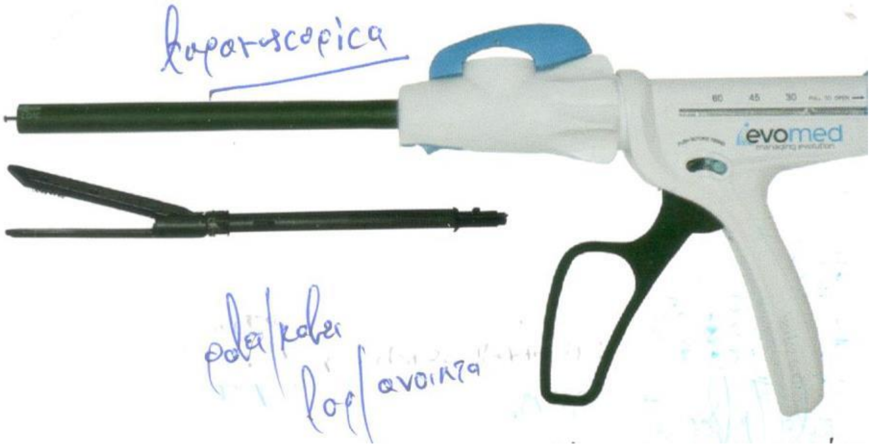

ανοικτα

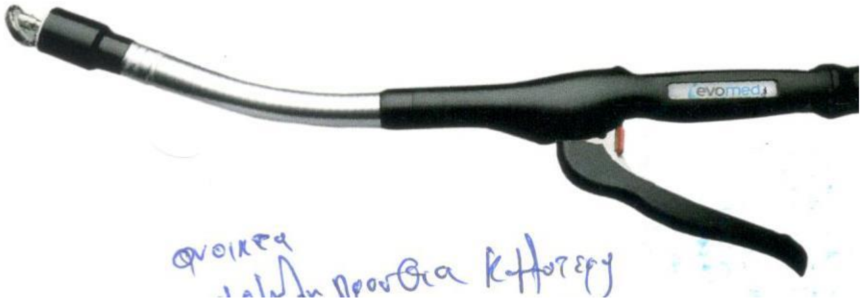

ανοικτα

με Σταθερή Κεφαλή V series

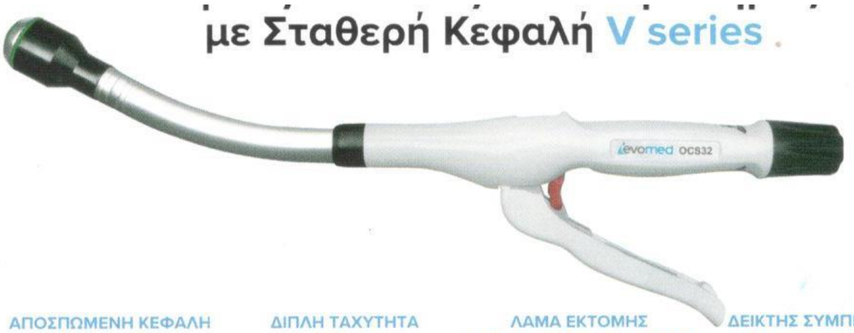

ΑΠΟΣΠΩΜΕΝΗ ΚΕΦΑΛΗ ΧΑΜΗΛΟΥ ΠΡΟΦΙΛ | ΔΙΠΛΗ ΤΑΧΥΤΗΤΑ ΑΝΟΙΓΜΑΤΟΣ | ΛΑΜΑ ΕΚΤΟΜΗΣ ΑΠΟ ΧΕΙΡΟΥΡΓΙΚΟ ΑΤΣΑΛΙ | ΔΕΙΚΤΗΣ ΣΥΜΠΙΕΣΗΣ ΠΛΗΡΩΣ ΟΡΑΤΟΣ

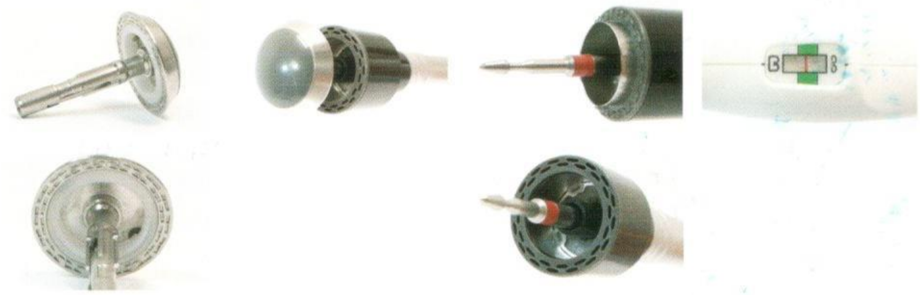

V series

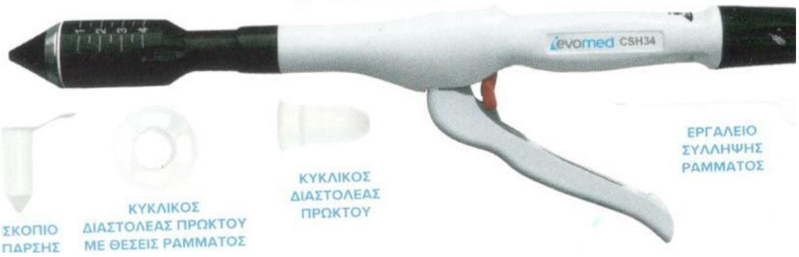

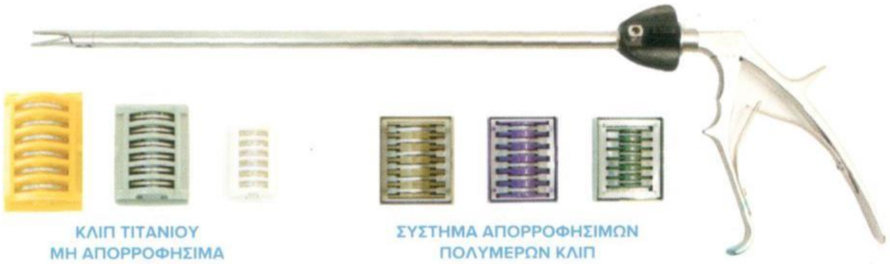

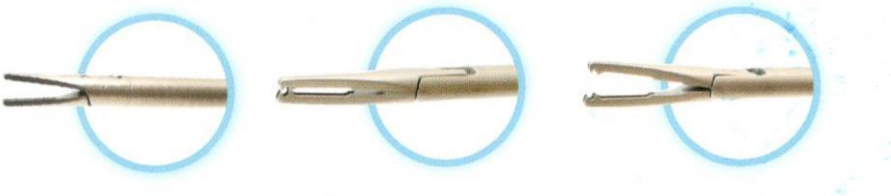

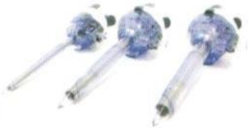

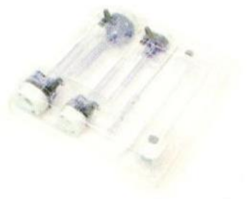

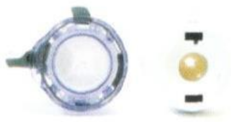

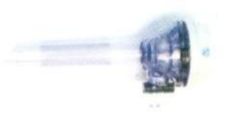

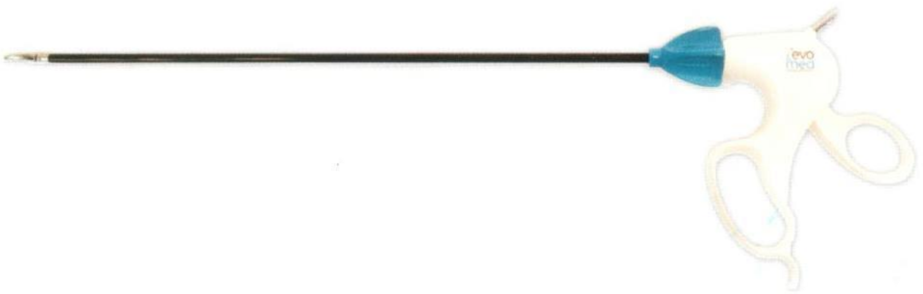
evo
med

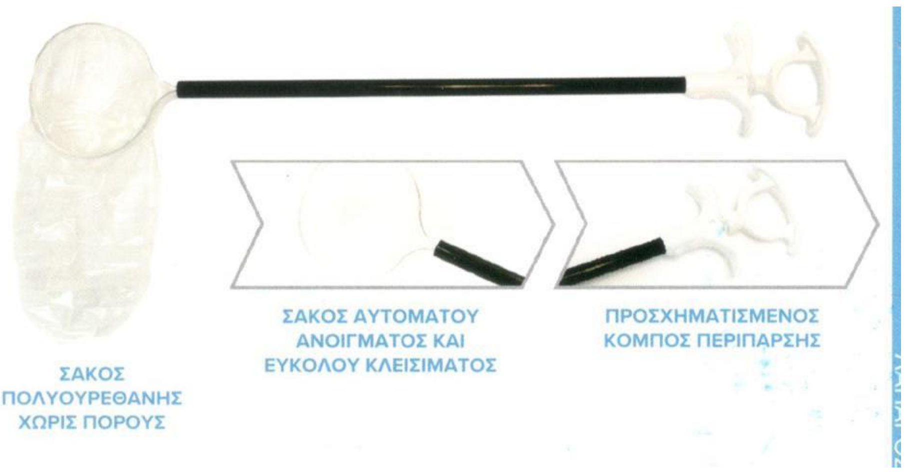
ΣΑΚΟΣ ΠΟΛΥΟΥΡΕΘΑΝΗΣ ΧΩΡΙΣ ΠΟΡΟΥΣ
ΣΑΚΟΣ ΑΥΤΟΜΑΤΟΥ ΑΝΟΙΓΜΑΤΟΣ ΚΑΙ ΕΥΚΟΛΟΥ ΚΛΕΙΣΙΜΑΤΟΣ
ΠΡΟΣΧΗΜΑΤΙΣΜΕΝΟΣ ΚΟΜΠΟΣ ΠΕΡΙΠΑΡΣΗΣ

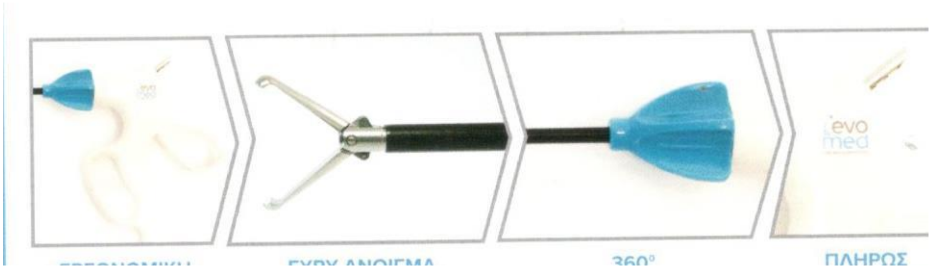
evo med
360°
ΠΛΗΡΩΣ
